# High proportion of horses in Germany positive for antibodies against Anoplocephalidae

**DOI:** 10.1186/s13071-026-07731-4

**Published:** 2026-10-28

**Authors:** Jennifer S. Schmidt, Jürgen Krücken, Jacqueline B. Matthews, Corinne J. Austin, Kirsty L. Lightbody, Jürgen Bartz, Georg von Samson-Himmelstjerna

**Affiliations:** 1 Institute for Parasitology and Tropical Veterinary Medicine, Freie Universität Berlin, Berlin, Germany; 2 Veterinary Centre for Resistance Research, Freie Universität Berlin, Berlin, Germany; 3 Austin Davis Biologics Ltd, Islip, Northants NN14 3FD UK; 4 Virbac Tierarzneimittel GmbH, Bad Oldesloe, Germany

**Keywords:** Equine, Cestoda, Epidemiology, *Anoplocephala perfoliata*, Infection risks, ELISA, Coproscopy

## Abstract

**Background:**

Tapeworms are often overlooked intestinal parasites in horses, although infections can potentially cause clinical disease. Antibody-detection–based diagnostic methods have significant advantages for estimating the proportion of horses positive for tapeworms, as some faecal routine examinations are often limited by low sensitivity.

**Methods:**

Data were obtained in the federal states Brandenburg, Bavaria, Lower Saxony, North Rhine-Westphalia, Saxony and Schleswig-Holstein. Coproscopy was performed using the Mini-FLOTAC technique (*n* = 970). Antibodies against the *Anoplocephala perfoliata* excretory-secretory 12–13 kDa antigen complex were measured using saliva- (*n* = 969) and plasma- (*n* = 957) based ELISAs (*n* = 923 paired samples). These data were combined with responses to a questionnaire to assess risk factors associated with tapeworm infection.

**Results:**

Tapeworm eggs were found in 0.7% (*n* = 7) faecal samples. Saliva-based ELISA testing identified positive tapeworm results in 20.1% of horses, with 60.4% of yards having at least one positive horse, while in the plasma-based ELISA, 11.0% of horses and 47.4% of yards were positive. Risk factor analysis using multivariable generalised estimating equations for binomial data, based on saliva ELISA results, showed that geographical origin was a significant determinant of infection risk. Horses from Brandenburg had the highest odds of testing positive (odds ratio [OR] 18.7; proportion 42.1%), while horses from Lower Saxony, Saxony and North Rhine-Westphalia also showed significantly higher odds (OR 4.1–12.4; proportion 17.4–32.2%) compared with Schleswig-Holstein (4.0%). Bavaria was the only state that did not differ significantly from Schleswig-Holstein (OR 2.7; proportion 9.0%). Another important factor was age: horses under 1 year (OR 0.41) or older than 5 years (OR 0.43) had significantly lower odds (*P* < 0.001) compared to horses aged 1–5 years. Strongyle faecal egg counts increased the odds of individuals being *Anoplocephala*-positive, with odds increasing 1.07-fold with every increase in egg counts by 100 (*P* < 0.001).

**Conclusions:**

The ELISA results show that horses in Germany are more frequently positive for Anoplocephalidae than indicated by Mini-FLOTAC. Strong regional differences in tapeworm saliva test positivity and the relatively low number of horses treated against tapeworms emphasise the importance of applying tapeworm diagnostics in control programmes.

**Graphical Abstract:**

**Figure:**
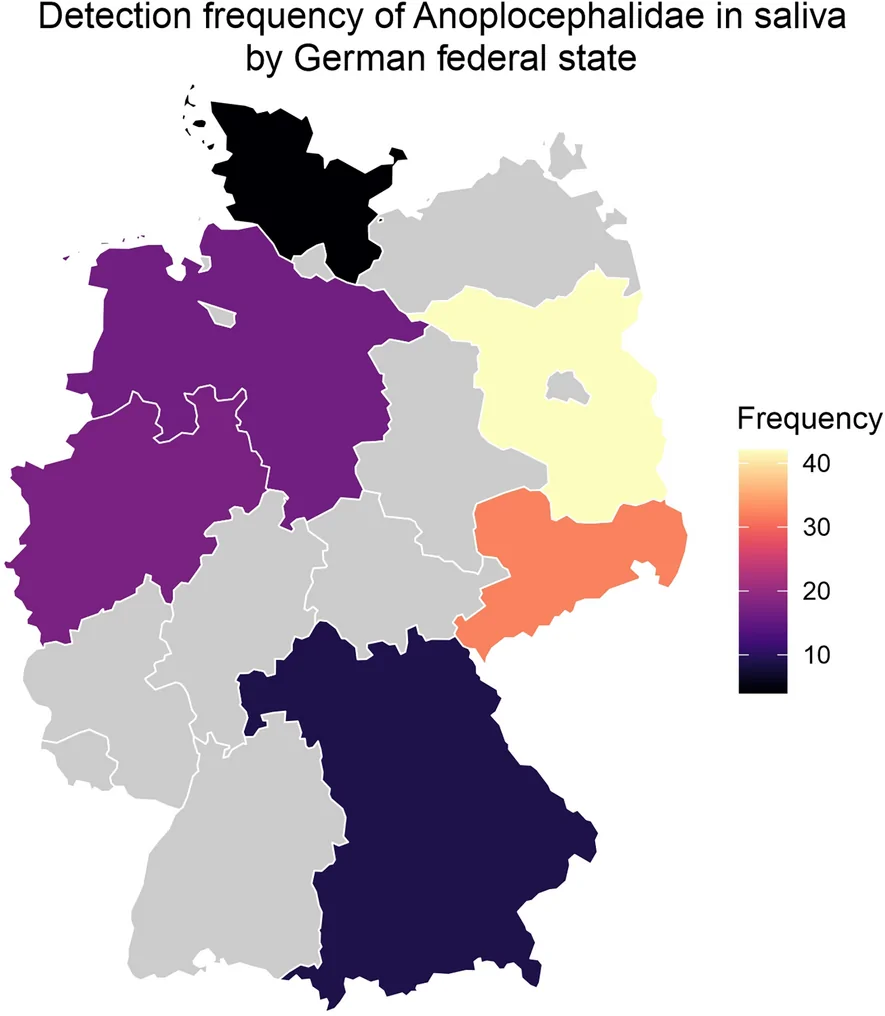

**Supplementary Information:**

The online version contains supplementary material available at https://doi.org/10.1186/s13071-026-07731-4.

## Background

The majority of horses in Germany have pasture access for at least part of the day, which will increase the risk for infection with gastrointestinal helminths [1, 2]. These infections typically consist of multiple types of parasites. In addition to the Cyathostominae, Strongylinae and *Parascaris* spp., tapeworms are common but often overlooked in horses. Equine tapeworm species that affect horses are *Anoplocephala magna*, *Anoplocephala perfoliata* and *Paranoplocephala mamillana*. While *A. magna* and *P. mamillana* live in the small intestine, the most commonly found species, *A. perfoliata*, is predominantly located in the caecum and ileum, often with accumulations around the so-called ileocaecal valve [3]. These tapeworms only infect horses, donkeys and other equids [4]. Infection occurs through the ingestion of cysticercoid-infected oribatid mites while grazing. These mites are an obligate intermediate host of the Anoplocephalidae, in which the infectious cysticercoid develops in 2–4 months [4].

There is wide variation in the reported prevalence of *A. perfoliata*, which may be influenced by ecological and seasonal differences in the abundance of the oribatid mites [5, 6]. However, pasture management factors and parasite detection methods are also likely to play an important role in the reported frequency of these parasites [7]. While horse-level frequencies of 28.5–75% have been found in post-mortem studies [8–10], studies using faecal examinations only report frequencies of 0.2–18.0% [1, 10–13]. Various studies have identified seasonality and horse age as important risk factors [5, 6, 8, 14]. Infection intensity in young horses can be higher compared to older horses, although horses of all ages appear to be susceptible to infection [15]. This suggests that even repeated infections do not lead to robust immunity [14, 16].

Infections with *A. perfoliata* are often inapparent, but can lead to emaciation, diarrhoea or colic. The degree of pathology has been shown to depend on the infection intensity [3, 17]. Some authors report significantly higher risk of spasmodic and ileal impaction colic in infected horses [18, 19]. This is likely due to the accumulation of tapeworms at the ileocaecal valve, leading to associated pathological lesions and impaired passage of ingesta [20]. Another influencing factor could be the reported pathological findings in the enteric nervous system, which might lead to intestinal motility issues [17]. In very rare cases, obturations, intussusceptions and perforations of the intestine can occur; without prompt surgical intervention, these conditions have a poor prognosis [19, 21].

There are various coproscopic, serological and molecular methods to detect equine tapeworm infections, but the sensitivity and specificity between methods vary considerably. Numerous flotation techniques have been described for detecting tapeworm eggs in faeces. However, for some methods, relatively low sensitivity has been reported [5, 22, 23]. Other methods, such as the Proudman and Edwards method and the Cornell-Wisconsin technique, have demonstrated better sensitivity and specificity for the detection of Anoplocephalidae eggs [24, 25]. However, these methods involve centrifugation and the Proudman and Edwards technique requires a larger amount of faeces.

Another approach is to detect specific antibodies through a blood- or saliva-based ELISA, which detect antibodies against the *Anoplocephala perfoliata* excretory-secretory 12–13 kDa antigen complex. These have been shown to have a sensitivity of 83–85% and a specificity of 78–85% [26–28]. However, potential cross-reactions are a possibility which needs consideration, as it was reported between *A. perfoliata* and *A. magna* [26]. The use of these tests has proven invaluable in sustainable worm control programmes, substantially reducing the number of tapeworm treatments compared with blanket deworming approaches [27, 29]. Further options are a coproantigen ELISA and various approaches via PCRs [30–33], but these have not been validated for routine diagnostic testing.

Only two cestodicidal compounds are currently available for the treatment of tapeworm infections in horses. These are praziquantel at a dosage of 1.0 mg/kg bodyweight (b.w.) orally, and pyrantel pamoate at an increased dosage of 13.2 mg/kg b.w. compared to the standard dose used for nematodes [34]. Importantly, for both drug classes, a reduction in treatment effectiveness was recently suggested in the US [35]. By promoting evidence-based tapeworm control using a combination of best management practices with targeted testing, overall parasite transmission can be reduced, thereby decreasing the number of horses requiring anthelmintic treatment. This strategy relies on understanding regional parasite prevalence and identifying key infection risk factors. For these reasons, this study aimed to collect new data on the proportion of horses positive for Anoplocephalidae in six different federal states of Germany through the use of Mini-FLOTAC and saliva- and plasma-based antibody testing and to assess the risk factors associated with test positivity.

## Methods

### Study design

The field study was carried out between October 2021 and December 2022 in six different federal states of Germany, chosen to cover different geographic regions. In 2021, sampling took place between October and December. The remaining samples were collected between June and December 2022. Included in the study were yards located in Bavaria, Brandenburg, Saxony, Lower Saxony, North-Rhine-Westphalia and Schleswig-Holstein. The sample size was determined based on findings from a previous study where approximately 30% of horses and 75% of yards were positive [1]. Horses sampled within the same yard were expected to have correlated infection statuses, and the yard was treated as the primary sampling cluster. The aim was to acquire 100 yards and sample approximately ten horses per yard, distributed across the six federal states.

In the previous study, approximately 30% of horses and 75% of yards were positive. Under a beta-binomial approximation, these proportions correspond to an intracluster correlation coefficient of approximately 0.31 for clusters of ten horses and a design effect of approximately 3.8. The planned sample of approximately 1,000 horses therefore corresponds to an effective sample size of approximately 262 independent observations. At an anticipated proportion of 30%, this provides an approximate 95% precision of ±5.5 percentage points for the overall animal-level prevalence.

With approximately 16–17 yards and 160–170 horses sampled per federal state, the corresponding effective sample size is approximately 44 horses per state. This provides an approximate state-specific precision of ±14 percentage points at an anticipated prevalence of 30%. For the anticipated yard-level prevalence of 75%, 16–17 yards per state provide an approximate precision of ± 20–21 percentage points. The study was therefore designed primarily to estimate overall prevalence, while providing less precise descriptive estimates for individual federal states.

Yards were contacted via email to provide them with information about the study, asking for voluntary participation. The email addresses were taken from the lists of the equestrian associations in the various federal states. Each yard that met the following criteria was allowed to take part: consent to faecal, saliva and blood sampling of 10 horses per yard, with no deworming in the last eight weeks and no strictly stabled horses. From each horse, it was aimed to collect a faecal, blood and saliva sample. For further analysis, the horses were categorised in the following groups: foals (up to 1 year), young horses (1–5 years old) and adults (over 5 years old). If owners were unable to specify the exact age of their horses, their age was assessed based on their teeth. Among the ten horses that were sampled, if available, at least one horse from each age group was included for each site. Grazing time was divided into the following categories: less than 4 h, 4–8 h and more than 8 h. At some yards, two or more of these categories were applicable.

Additionally, a questionnaire (Additional file 1: Text S1. Questionnaire) was completed by the yard owner, manager or one of the horse owners. Questions covered the age, sex, date and last used anthelmintic of the horses that participated and, at the yard level, about deworming and grazing management practices, paddock size, and the number of horses on the premises.

### Faecal analyses using Mini-FLOTAC

From all horses, faecal samples were obtained rectally or collected from the ground immediately after defaecation. The collected samples were stored in 5-l buckets, one for each horse, and labelled. Each sample was thoroughly blended in the bucket, and 5 g were weighed into a 50-ml conical centrifugation tube. Since it was not feasible to perform the faecal examination on site, samples were preserved (see below) during transportation to the laboratory. This prevented larval development in nematode eggs and allowed faecal egg count (FEC) examinations for the strongyles, which were also within the scope of this project. All 5 g of the sample were mixed with 1.5% Lugol’s iodine solution in a ratio of 1:2 (weight/volume). Lugol’s iodine was prepared as previously described by Demeler et al. [36]. At the laboratory, samples were centrifuged at 1500 × g for 2 min, and the supernatant containing most of the iodine solution was removed. FEC assessment was made using Mini-FLOTAC devices [37] with small modifications [22]. For each sample, two chambers of the Mini-FLOTAC device were filled and counted. The obtained raw egg counts were multiplied by five to obtain the eggs per gramme (epg) count in faeces.

### Saliva- and plasma-based ELISAs

Saliva samples were collected from 969 horses with Equisal® collection kits and analysed in the EquiSal® Tapeworm Saliva Test [26] service provided by Austin Davis Biologics Ltd. (Northamptonshire, UK). Owners were asked to ensure that their horses did not receive any food and water or perform any heavy work for at least 30 min prior to sampling to ensure the optimal test conditions. The swabs, contained in the kit, were placed in preservative solution after sample collection, labelled and frozen at − 20 °C within 2 h after sampling. Samples were shipped frozen and tested following thawing. Based on the obtained saliva scores from the test, a diagnosis of Low (< − 0.09), Borderline (− 0.09–0.6) or Moderate/High (> 0.6) was reported for each sample. Horses with a Borderline or Moderate/High saliva score were considered positive and a yard was categorised positive if at least one horse reported a Borderline or Moderate/High result. If samples contained insufficient total IgG(T) and were deemed to be too diluted, they were excluded from further analysis. There are different reasons for this; for instance, the horses might have salivated too much, received food just before sampling or not enough saliva was collected [26].

Blood plasma samples were collected from 957 horses and tested with the Tapeworm Blood Test [26] by Austin Davis Biologics Ltd. (Northamptonshire, UK). From each horse, 10 ml blood was collected under sterile conditions in an EDTA tube and cooled until centrifugation. The blood was centrifuged to obtain the plasma at 2000×g for 10 min within 2 h after collection and aliquoted into sterile 2-ml tubes. The samples were frozen and stored at − 20 °C and shipped frozen. Plasma scores with a cut-off value of < 2.7 were reported as Low, scores between 2.7 and 6.3 were reported as Borderline and scores above 6.3 were reported as Moderate/High. Moderate/High and Borderline scores were considered positive.

### Data analyses

The collected data were first documented on the questionnaires in paper form and later transferred to an Excel spreadsheet. All statistical analyses were performed with R version 4.3.3 using R Studio 2024.04.02 Build 764 for Windows.

The 95% confidence intervals (CI) for the proportions were calculated with package samplingbook version 1.2.4 with the function Sprop() using a correction for the known finite population size on individual yards. For federal states or the complete dataset, where the population size was unknown, the population size was set to indefinite and 95% CIs were calculated as Wilson score intervals. Differences between frequencies in different federal states were calculated using the mid-p exact test as implemented in the epitools version 0.5–10.1 tab2by2.test() function. Correction for multiple testing was performed using p.adjust() from the stats package (version 4.3.3), applying the Holm correction.

Generalised estimating equations (GEE) were used for the risk factor analysis, with yard included as the random effect variable, to identify variables that have an influence on the odds for the horses to test positive in the tapeworm saliva test. The risk factor analyses were performed with the saliva test, as the specificity of this is slightly higher compared to the plasma ELISA [26]. Another factor to consider is that the antibodies can be detected in the blood up to six months after treatment [26], with a recommended application of the plasma test at least four months after treatment [26, 38]. In comparison, antibodies detected in saliva decrease more rapidly post-treatment, and follow-up testing can be performed from 12 weeks after treatment [29]. The number of horses that tested positive in the faecal examination was too low for risk factor assessment. The models were calculated with the function geeglm() from the geepack package version 1.3.12. All variables were initially analysed in a bivariable analysis. Then, variables that had a *P* ≤0.2 were included in a multivariable analysis, and the initial model was stepwise reduced by elimination of variables to obtain a better (lower) quasi-likelihood information criterion (QIC).

A Pearson correlation index was calculated in GraphPad Prism Version 5.03. to evaluate the correlation between the plasma and saliva scores. To evaluate the inter-rater agreement between both tests, Cohen’s kappa (κ) was calculated in R with the package DescTools version 0.99.58 with the function CohenKappa().

The Kruskal–Wallis test was used to assess significant differences in yard-level saliva test frequencies across federal states. Post hoc pairwise comparisons were then performed using Dunn’s Multiple Comparison Test to identify which states differed significantly. Both analyses were conducted in GraphPad Prism, version 5.03. All graphs were plotted with GraphPad Prism Version 5.03.

## Results

### General data on horse and yard levels used for risk factor analyses

The general data of the study population are provided in Table 1. Out of the 970 horses from 96 yards, 178 horses were from Brandenburg, 121 from Saxony, 164 from Lower Saxony, 177 from North-Rhine-Westphalia, 178 from Schleswig-Holstein and 151 from Bavaria. Differences in horse/yard numbers between federal states were due to different recruitment success between states. Of these horses, 3.4% belonged to age group 1 (foals under 1 year), 16.6% to age group 2 (1–5 years) and 80.0% to age group 3 (adult horses over 5 years). The owners of 11 horses from 2 stables were not able to provide age data, and therefore, the age of the horses was estimated based on their teeth. Out of the 970 horses, 47.4% were mares, 47.2% geldings and 5.4% stallions.

**Table 1:** General data of the study on the horse level

Parameter	Value	Number of horses for this variable
Age (median; range in years)	12.0 (0.2–38.0)	959
Age group		970
Foals (< 1 year) (%)	3.4	33
Young horses (> 1-5 years) (%)	16.6	161
Adult (> 5 years) (%)	80.0	776
Sex		970
Mares (%)	47.4	460
Geldings (%)	47.2	458
Stallions (%)	5.4	52
Samples collected		
Faecal samples (%)	100	970
Saliva samples (%)	99.9	969
Plasma samples (%)	98.7	957
Horses sampled per yard (number; median, range)	10 (8–12)	970
Active substance for last treatment		970
Fenbendazole (%)	13.5	131
Pyrantel (%)	29.3	584
Moxidectin (%)	13.1	127
Ivermectin (%)	33.7	327
Doramectin (%)	1	10
Ivermectin + praziquantel (%)	6	58
Moxidectin + praziquantel (%)	3	29
Not treated ≥ 1 year (%)	0.4	4
Period between last anthelmintic treatment and sampling in weeks (median, mean, range)	20; 20.8 (6–51)	970
Pasture access (%)	100	961
Number of horses per yard (median, range)	37 (9–200)	961
Foals present (%)	43.2	961
Positive for GIN in faecal examination		970
Foals < 1 year (%)	75.8	33
Young horses (> 1–5 years) (%)	71.4	161
Adult (> 5 years) (%)	47.7	776
Pasture time per day		961
< 4 h	19.4	186
Between 4 and 8 h	23.8	229
> 8 h	33.8	325
2 or more options applicable in the yard	23.0	221
Faeces removal from pasture		961
Regularly	31.1	299
No cleaning	64.7	622
Partially	4.2	40

*GIN* = gastrointestinal nematodes

In the faecal examination, using the Mini-FLOTAC, gastrointestinal strongyles eggs were detected in 47.4% of the horses and in 95.8% of the yards (92 out of 96 positive). Among the age groups, the proportion of positive tested horses was 75.8% in group 1 (foals), 71.4% in group 2 (juveniles), and 47.7% in group 3 (adults). *Parascaris* spp. eggs were found in 0.9% of the samples and *Oxyuris equi* in 0.2%.

Grazing time varied across the horse yards: in 18 yards, horses grazed less than 4 h per day; 23 yards recorded 4–8 h; 32 yards exceeded 8 h daily; 22 yards reported mixed durations and one yard provided no information. Of the 95 out of 96 yards that answered the questionnaire, 29 (31.1%) stated that they regularly remove faeces from the pasture, while 62 (64.7%) never removed faeces, four partially removed faeces (4.2%).

All yard owners reported that all horses were dewormed at least once per year. Forty-three yards dewormed once or twice annually, 38 yards three times, and 14 yards at least four times per year; one yard provided no information. Foals and young horses were typically dewormed at least four times annually, even when adult horses were treated less frequently. While 12 yards reported selective deworming, only four met the criteria for selective treatment based on faecal examination results and individualised treatment decisions. Even these yards dewormed all horses at least once yearly.

Praziquantel was administered to 9% of the horses at their last anthelmintic treatment prior to sampling. However, this was always in a combination product including moxidectin (3%) or ivermectin (6%). In addition, 29.3% of the horses were last treated with pyrantel, but only at the standard dosage of 6.6 mg/kg b.w. One-third of the yard operators stated that they change the horses’ pasture after deworming.

### Faecal detection of Anoplocephalidae eggs

Of the 970 faecal samples tested with Mini-FLOTAC, only seven horses were positive for Anoplocephalidae eggs; resulting in a proportion on horse-level of 0.7% and a yard-level proportion of 4.2% (Table 2). Three tapeworm egg-positive horses were from Brandenburg (all from yard 6), two from Saxony (both yard 19), one from North-Rhine-Westphalia (yard 28) and one from Bavaria (yard 76). The epg recorded was between 5 and 35. Six of the seven horses belonged to age group 3 and one to age group 2. All animals that tested positive for Anoplocephalidae eggs were also positive for gastrointestinal nematodes (GINs) with an epg ranging between 30 and 745 strongyle eggs. Four of the horses that tested positive for Anoplocephalidae eggs were last dewormed with pyrantel, two horses with fenbendazole and one horse with a combination of moxidectin and praziquantel.

**Table 2:** Proportion of positive Anoplocephalidae test results obtained through different diagnostic methods

	Horse level		Yard level	
Method	n/N^a^	Proportion of positive horses in % (95% CI)^b^	n/N^a^	Proportion of positive yards in % (95% CI)^b^
Mini-FLOTAC	7/970	0.7 (0.4 – 1.5)	4/96	4.2 (1.6 – 10.3)
Saliva-based ELISA^c^	188/935	20.1 (17.7 – 22.8)	58/96	60.4 (50.4 – 69.6)
Plasma-based ELISA^d^	105/957	11.0 (9.2 – 13.1)	45/95_e_	47.4 (37.6 – 57.3)

^a^n = positive tested horses/yards in each category; N= total number of horses/yards in this category

^b^Confidence intervals calculated as Wilson score intervals

^c^Samples with low total IgG(T) concentration were excluded

^d^For some horses, no blood samples could be obtained

^e^One yard was excluded since blood could only be taken from one horse in this stable

### Saliva-based ELISA

In total, 969 saliva samples were obtained and analysed from 96 yards in the tapeworm saliva test. Of these, 34 samples were excluded due to low total IgG(T) in the samples. Overall, 188/935 horses tested positive in the saliva-based ELISA, which resulted in a horse-level proportion of 20.1% (95% CI 17.7–22.8%) and a yard-level proportion of 60.4% (95% CI 50.4–69.2%). Of the samples tested, 6.1% (95%CI 4.7–7.6) were diagnosed Borderline and 14.0% (95%CI 11.9–16.4) Moderate/High.

**Fig. 1:**
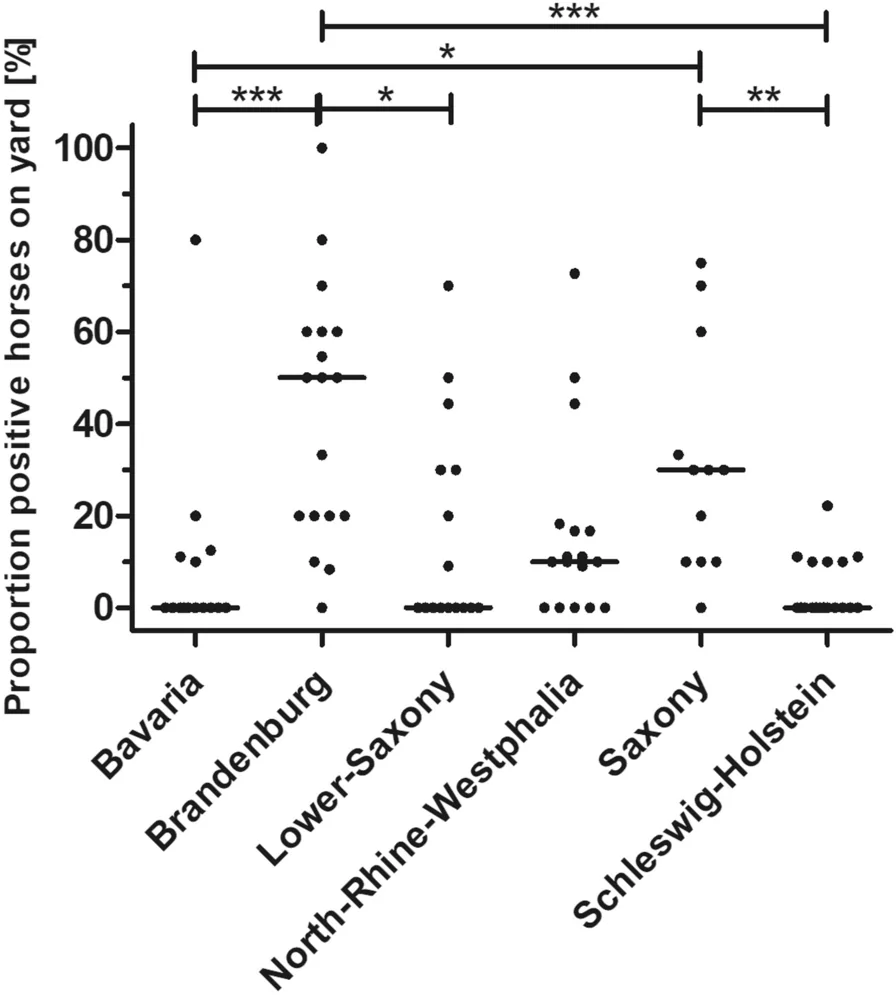
Individual horse–level saliva-based ELISA frequency across horse yards in six German federal states. Each point represents a yard. The Kruskal-Wallis followed by Dunn’s multiple comparison test were used to compare the frequencies of the saliva-based ELISA on yard level between different federal states. * P < 0.05; ** P < 0.01; *** P < 0.001

Table 3 shows the proportion of positive tested horses through the saliva-based ELISA for the six German federal states included in this study. Proportion of positive horses varied between the different federal states (Table 3), with the highest in Brandenburg and Saxony, and the lowest in Schleswig-Holstein and Bavaria. Pairwise differences were in most cases significant, which were evaluated with the mid-p exact test after correcting for multiple testing using the Holm method. Frequencies were also calculated at the yard level (Additional file 2: Table S1). Kruskal–Wallis analysis showed that there were significant differences (*P* < 0.001) in yard-level proportion of positive horses among the six federal states. Dunn's multiple comparison test was used to analyse the significance of yard-level frequencies between the federal states (Fig. 1).

**Table 3:** Proportion of positive horses according to the saliva-based ELISA in different federal states

Federal state	n/N^a^	Proportion of positive horses in % (95% CI)^b^
Brandenburg	75/178	42.1 (35.1–49.5)^c^
Saxony	39/121	32.2 (24.6–41.0)^c^
Lower Saxony	25/151	16.6 (11.5–23.3)^d,e^
North-Rhine-Westphalia	29/167	17.4 (12.4–23.8)^d,f^
Schleswig-Holstein	7/174	4.0 (2.0–8.1)^g^
Bavaria	13/144	9.0 (5.4–14.8)^e,f,g^

^a^n = positive testes horses in each category; N = total number of horses in this category excluding those with low protein concentration

^b^Confidence intervals calculated as Wilson score intervals

^c^^−^^g^Federal states that do not share at least one index are significantly different from each other in a mid-p exact test after correcting for multiple testing using the Holm method (*P* < 0.05)

### Plasma-based ELISA

Plasma samples were obtained from 957 of the 970 horses. Of these, 105 tested positive in the plasma-based ELISA, demonstrating an overall frequency of 11.0% (95% CI 9.2–13.1%) (Table 2). In total, 4.6% (95% CI 3.4–6.1%) were diagnosed as Borderline and 6.4% (95% CI 5.0–8.1%) as Moderate/High. The proportion on yard level was 47.4% (95% CI: 37.6–57.3).

### Comparison between Mini-FLOTAC examination, saliva- and plasma-based ELISAs

In total, 923 matching samples with faecal, saliva and plasma results were compared. All horses that tested positive in the faecal examination had a diagnosis of Moderate/High in both the saliva- and plasma-based ELISAs.

**Fig. 2:**
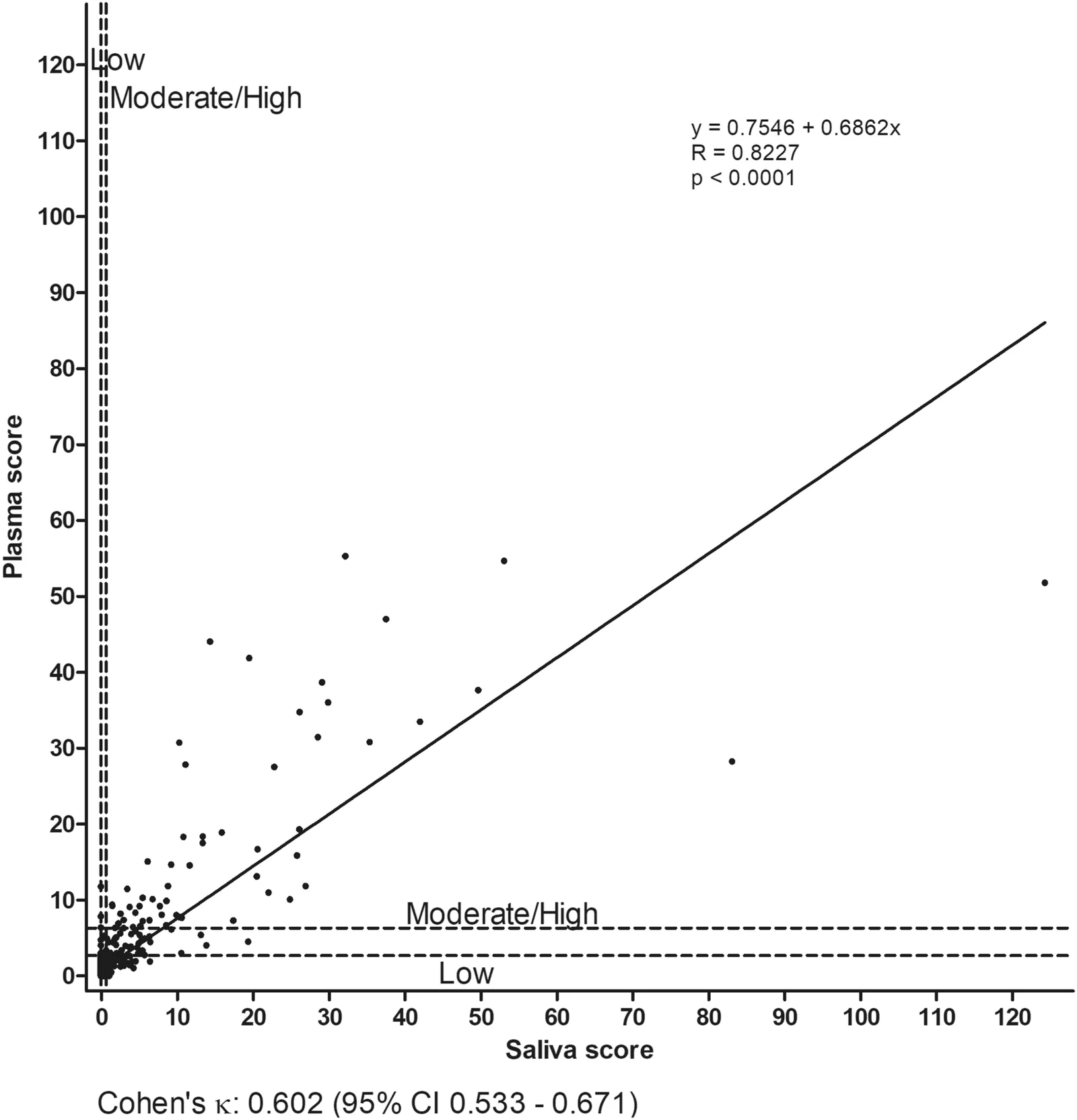
Correlation of saliva- and plasma-based ELISA scores for 923 matching pairs. The dashed lines indicate the cut-off values for the different Low and Moderate/High groups. In between the dashed lines are the Borderline samples. Cut-off values for the saliva-based ELISA were: < -0.09 Low; -0.09 – 0.61 Borderline; ≥ 0.62 Moderate/High; cut-off values plasma-based ELISA were: < 2.7 Low; 2.7 – 6.3 Borderline; > 6.3 Moderate/High

Of the matching samples, 187 (20.3%; 95% CI 17.8–23.0%) were positive in the saliva-based ELISA and 104 (11.3%; 95% CI 9.4–13.5%) in the plasma-based ELISA. Additional file 2 Table S1 shows the observed saliva and plasma ELISA frequency for each yard. A Pearson correlation was calculated and showed a significant correlation of both scores (*P* < 0.001) and a coefficient of *r* = 0.823, which indicates a strong positive correlation between the saliva and plasma scores (Fig. 2). The mid-p exact test indicated that significantly more animals were positive in the saliva-based ELISA (20.1%) than in the plasma-based ELISA (11.0%) (*P* < 0.001). For 824 samples, both ELISAs resulted in the same binomial assignment as positive (96 samples) or negative (728 samples). There were 91 samples that were positive in the saliva-based ELISA (36 Moderate/High and 55 Borderline) but negative in the plasma-based ELISA, and eight samples that were positive in the plasma-based ELISA (3 Moderate/High and 5 Borderline) but Low in the saliva-based ELISA. From these data, a Cohen’s κ inter-rater agreement between both tests of 0.602 (Fig. 2) was calculated, which revealed a moderate agreement between both tests.

### Risk factor analyses for the saliva-based ELISA results obtained by logistic generalised estimating equations

Table 4 shows all odds ratios of the variables, which were initially included in bivariable risk factor analyses as well as the final multivariable model after optimisation.

**Table 4:** Risk factor analysis models with odds to be tested positive in the saliva-based ELISA for different variables

			Bivariable models			Multivariable model		
Variable	N^a^	Frequency positives (%)	OR^b^	95% CI^c^	P	OR	95% CI	P
Age group	935							
<1 years	32	5 (15.6)	0.40	0.17 – 0.98	0.046*	0.41	0.15–1.10	0.077
1–5 years	159	50 (31.4)	1			1		
>5 years	744	133 (17.9)	0.47	0.29–0.77	0.003*	0.43	0.26–0.71	<0.001*
Change of pasture	926							
Never	228	27 (11.8)	1					
Partly	19	7 (36.8)	4.34	1.08–17.51	0.039*			
Regular	679	154 (22.7)	2.18	1.02–4.68	0.045*			
Drug against tapeworm	935							
Praziquantel (PZQ)	81	9 (11.1)	0.47	0.17–1.34	0.159			
Pyrantel (PYR)	277	55 (19.9)	0.88	0.49–1.62	0.698			
None	577	124 (21.5)	1					
Epg gastrointestinal strongyles/100	935	n.a.	1.01	1.05–1.15	<0.001*	1.07	1.02–1.14	<0.001*
Federal state	935							
Brandenburg	178	75 (42.1)	17.37	7.09–42.55	<0.001*	18.7	7.40–47.12	<0.001*
Bavaria	144	13 (9.0)	2.37	0.54–10.67	0.253	2.66	0.63–11.23	0.182
Lower Saxony	151	25 (16.6)	4.73	1.60–13.97	0.005*	4.07	1.36–12.25	0.012*
North-Rhine-Westphalia	167	29 (17.4)	5.01	1.80–13.95	0.002*	4.97	1.76–14.03	0.002*
Saxony	121	39 (32.2)	11.35	4.26–30.23	<0.001*	12.35	4.46–43.18	<0.001*
Schleswig-Holstein	174	7 (4.0)	1			1		
Fertilisation with faeces	926							
No	733	146 (19.9)	1					
Yes	193	42 (21.8)	1.12	0.53–2.37	0.771			
Pasture time	926							
Access up to 4 h	175	33 (18.9)	0.69	0.26–1.84	0.461			
Access between 4 and 8 h	220	40 (18.2)	0.66	0.30–1.45	0.302			
Access over 8 h	318	80 (25.2)	1					
2 or more applicable	213	35 (16.4)	0.58	0.25–1.34	0.206			
Size pasture in hectare	926	n.a.	1.0	0.99–1.0	0.755			
Size of stable	926	n.a.	1.0	0.99–1.0	0.537			
Pasture cleaning	926							
Never	608	156 (25.7)	1					
Partly	36	2 (5.6)	0.17	0.06–0.51	0.002*			
Regular	282	30 (10.6)	0.34	0.15–0.51	0.012*			
Times deworming	918							
Once per year	76	10 (13.2)	1					
Twice per year	334	92 (27.5)	2.51	0.79–7.97	0.119			
Thrice per year	368	65 (17.7)	1.41	0.45–4.49	0.555			
>4 Times per year	140	21 (15.0)	1.16	0.32–4.12	0.816			

n.a. = not applicable

N^a^ = number of horses in this category

OR^b^ = odds ratio

CI^c^ = confidence interval

^*^Statistically significant

In the bivariable analysis, there were strong differences regarding the odds of being tapeworm saliva test positive for horses originating from different federal states. Horses from Brandenburg, Saxony, Lower Saxony and North-Rhine-Westphalia had significantly higher odds of testing positive in comparison to the reference, Schleswig-Holstein. Foals under one year and adult horses over five years had lower odds for positive test results compared to horses between one and five years. Both groups showed comparable odds ratios between 0.4 and 0.5 in comparison to the intermediate age group.

An increasing strongyle faecal egg count was also associated with significantly higher odds of a positive ELISA result. For every increase in epg by 100, there was an increased odds of 1.01 to be tested positive in the saliva ELISA.

Conducting pasture hygiene measures was associated with significantly lower odds of a positive tapeworm saliva test result, with regular or occasional cleaning reducing risk compared with no pasture hygiene. The effect of partial hygiene measures was stronger than that of regular hygiene measures; however, all horses subjected to regular paddock hygiene measures came from only four yards.

Both regular and partial pasture changes were associated with statistically significantly higher odds of positive test results, compared to never changing. Faeces removal was reported less frequently when paddocks were changed regularly or partly (27.4% of yards) than on yards where paddocks were never changed (55.7%, *P* < 0.001, mid-p exact test). Daily pasture time did not have a significant effect on the odds of a positive antibody test, although horses grazing up to 4 h or between 4 and 8 h per day showed (non-significantly) lower odds compared with those grazing over 8 h daily.

Horses that were treated the last time before sampling with praziquantel had 50% lower, but not significantly different, odds (*P* = 0.159) for being positive in the saliva-based ELISA in comparison with a treatment without an active agent against tapeworms. Additional variables without an effect in the bivariable models were the number of horses on the premises, size of pasture (in hectares), annual deworming frequency, daily pasture time and fertilisation of pasture with faeces. Pyrantel was administered in all horses only at a dose of 6.6 mg pyrantel per kg body weight, which has only limited efficacy against tapeworms, and none were treated with 13.2 mg/kg. Pyrantel had no significant effect (*P* = 0.698).

In the multivariable model, all variables with *P* <0.2 in the bivariable models were initially included: epg of strongyle nematodes, annual deworming frequency, pasture time, federal state, the use of cestocidal anthelmintics (praziquantel), pasture hygiene practices and age group. After stepwise optimisation of the model by eliminating variables to improve the QIC, the final model only contained the following variables: federal state, age group and presence of strongyle eggs in the faeces (Table 4).

In the multivariable model, the origin of the horses was still an important variable as horses from Brandenburg, Saxony, Lower Saxony and North-Rhine-Westphalia had significantly higher odds of being tested positive in the saliva ELISA test than horses from Schleswig-Holstein, which was chosen as the reference. A positive FEC for strongyles in the Mini-FLOTAC analysis was also associated with significantly higher odds for being positive in the tapeworm saliva test. Foals under 1 year and adult horses over 5 years had lower odds for tapeworm-positive saliva testing in comparison to horses aged between 1 and 5 years.

## Discussion

In this study, frequency data for equine tapeworm infection for direct detection of parasite stages using coproscopy and indirect detection of antibodies against the parasite in German horses were obtained using three different detection methods, which allows a comparison of the results. The FECs were obtained with the Mini-FLOTAC method. A previous study has shown no significant difference in the detection of the eggs between the Mini-FLOTAC and the combined sedimentation–flotation [1, 22, 39], and similar results were also reported by other authors [22, 39]. Other methods such as the Proudman and Edwards or the Cornell-Wisconsin technique, which were developed for the detection of tapeworm eggs report a higher sensitivity [25, 40], had shown a better egg recovery in a study with direct comparison between the Mini-FLOTAC and these techniques [24]. However, both techniques require, compared to the Mini-FLOTAC method, a higher amount of faeces, centrifugation and a notably longer processing time. Since this study also intended to collect data regarding the frequency of nematode infections, the Mini-FLOTAC was selected as a compromise to allow detection and quantification of both nematode and cestode eggs. Tapeworm eggs were detected in 0.7% of the samples and in 4.2% of the yards. The proportion of positive tested horses in this study is in the lower range of the results of previous studies conducted in Germany, which reported prevalences of 0.2% using a modified McMaster method [13] and 2.0%, 3.0% and 18% using combined sedimentation/flotation [10–12]. Noteworthy, the results are more similar to recent data, with 0.6% horse level and 6.3% yard-level frequency found by Jürgenschellert et al. [1] in Brandenburg and Berlin. In comparison, post-mortem studies report considerably higher prevalences of 28.5 to 38% [8, 10], although Beelitz and Gothe [10] also reported an proportion of positive horses of 18% by analysing faecal samples from necropsied horses using the combined sedimentation–flotation method. These differences are likely due to a variety of factors such as season of sampling, health status, management and hygiene practices of the horses assessed and the often reported low sensitivity of many FEC analyses including standard flotation methods for detecting tapeworm infections [4, 24, 41–43].

Compared to the frequency data from the Mini-FLOTAC examination, saliva- and plasma-based ELISA proportions at the horse-level were 20.1% and 11.0%, respectively. Higher frequencies in ELISAs based on detection of antibodies (indirect detection) can be partially explained by the difference between infection (direct parasite detection by coprocopy) and exposure (indirect detection via antibodies). The tapeworm ELISAs used in this study were validated against post-mortem worm counts to establish the diagnostic thresholds applied [26]. These tests seem to offer a sensitivity advantage over the Mini-FLOTAC for detecting *A. perfoliata.* Since the latter is associated with lower sensitivity and might therefore detect a lower number of positive horses, compared to coproscopic methods, which show a better egg recovery for tapeworm infections, such as the Proudman and Edwards method, it remains unclear if serology in general has a better sensitivity than coproscopy.

To compare the two ELISAs, a Pearson correlation coefficient was calculated, indicating a strong positive correlation (*R* = 0.783). With a Cohen’s κ value of 0.602, a moderate agreement between both tests was indicated, with significantly more positive test results in the saliva-based ELISA compared to the plasma-based ELISA (*P* < 0.001). Similar frequencies were obtained in other studies with the saliva- and plasma-based ELISAs [1, 39]. The differences between Mini-FLOTAC diagnosis and serology are probably in part due to the above-mentioned lower sensitivity of the Mini-FLOTAC as similar discrepancies have been previously observed [1, 39]. In addition, a positive result on the ELISA tests does not necessarily indicate an active infection but could also show a previous exposure with persisting antibodies after treatment. Tapeworm-specific antibodies present in saliva at the time of treatment can be detected for up to 12 weeks afterwards [29], although it has been shown in one study that 70% of horses fall below the treatment threshold by 5 weeks [26]. In contrast, antibodies in blood can persist for 4–6 months post-treatment [26, 38]. This might lead to an overrepresentation of positive test results, and serological results should be interpreted with caution. However few horses in the study were treated with cestocidal anthelmintics within these timeframes, in particular the 12-week window. Differences in some of the results between the two ELISAs have also been reported previously [1, 7, 39]. Despite longer persistence of antibodies in blood than in saliva, the current study found more positive results with the saliva-based ELISA than the plasma-based ELISA. A possible explanation might be that the sensitivity of the saliva-based ELISA is higher compared to the plasma-based ELISA as a better correlation between tapeworm burden as assessed post mortem and the test results was reported previously [26]. This might follow the reported theory that the detected antibodies in the saliva-ELISA are mucosal IgG(T), which are produced in the mucosal wall of infected horses and in the salivary gland [26, 44].

Most horses that received praziquantel as their last anthelmintic treatment prior to sampling (*n* = 87) had not been dewormed for 13–48 weeks, with the exception of two horses treated eight weeks before sampling. Among these praziquantel-treated horses, nine tested positive in the saliva-based ELISA, including five with Moderate/High scores and four with Borderline scores. In comparison, only five of these horses were positive in the plasma-based ELISA (four Borderline, one Moderate/High). One horse with a Moderate/High saliva score tested negative in the plasma test. However, the plasma score of 2.63 was just below the Borderline cut-off. Since pyrantel treatment had an even smaller, non-significant effect in the bivariable model than praziquantel, it is unlikely that recent treatment with this drug affected the results.

The frequency of positive horses, based on saliva testing, in the individual federal states differed noticeably, with low proportion of positive horses in Bavaria (9%) and Schleswig–Holstein (4%) and high proportions in Brandenburg (42.1%) and Saxony (32.2%), and the individual frequencies in the different yards followed the same trend. These differences might be due to various influencing factors. Seasonality is an often-described phenomenon associated with tapeworm infections with a prevalence peak in the winter months [5, 8, 14]. This might have led to an overestimate of frequencies in Brandenburg in the present study as the samples from Brandenburg were mainly collected at the end of 2021, while the samples from the federal states with low frequencies, such as Schleswig-Holstein and Bavaria, were collected from August to October 2022. Jürgenschellert et al. [1] found a high prevalence of 29.5% using the saliva-based ELISA for Brandenburg and Berlin, with a collection of the samples in 2017/2018, which is somewhat lower than the proportion reported in the current study. However, the tested yards in Saxony also showed a relatively high proportion of 32.2%, although Saxony was the only federal state in which samples were collected over the whole grazing season. In all other federal states, except Brandenburg, samples were collected in one to two week time windows due to logistic limitations, which could have led to an over- or underestimation of the proportion in some states. Therefore, seasonality does not appear to be the only factor that contributes to the differences in proportion of positive tested horses among the federal states.

The differences between data from Brandenburg/Berlin 2017/2018 and the higher proportion recorded in the present study in Brandenburg might partly be explained by the fact that the latter were collected after a very rainy summer 2021 [45]. This might have created a comparatively humid environment, favouring the development and survival of the oribatid mites serving as intermediate hosts. Moreover, the drought in 2017/2018 might have led to a higher proportion of hay in the feed and lower grass intake of the horses, further reducing the infection risk. In comparison, 2022 was very dry and hot in large parts of Germany, which may have had a negative impact on the oribatid mite populations and intake of fresh grass. This could be the reason for the lower frequency in federal states such as Schleswig-Holstein and Bavaria, where all samples were taken in the summer of 2022. However, the climate in large parts of Saxony is considered very dry with lower mean annual rainfall [46] compared to the other federal states (e.g. SH, Bavaria). Further variables such as local husbandry conditions, on-site paddock hygiene measures and the use of particular anthelmintics may also influence tapeworm frequency. The findings suggests that the observed differences are most likely the result of multifactorial variables that were not recorded using the study questionnaire. This is a limitation of the current study and the reason why differences between federal states were so large remains unclear.

According to the bi- and multivariable risk factor analyses, three factors were found to be associated with significant differences in the frequency of tapeworm infections, and some tendencies could be seen. The odds for positive saliva-based ELISA results differed between the federal states. Horses from Brandenburg, Saxony, Lower Saxony and North-Rhine-Westphalia had significantly higher odds for positive saliva test results in comparison to Schleswig-Holstein. The only variable associated with lower odds of being seropositive in the final risk assessment was the age group, as foals under a year and adult horses over 5 years had lower odds, which has been observed previously [47]. Other recent studies also reported lower odds for horses with increasing age [1, 39]. That foals under one year of age have lower odds for infections was previously described by Nilsson et al. [14]. A possible explanation could be that half of the foals were under six months of age at the sampling, so that a large proportion of their feed was milk, which lowered the infection risk. Another explanation could be that most foals came from relatively small premises with high standards of hygiene in the area of foal husbandry. As the number of foals in this study is very small in comparison to the other age groups, the results should be treated with caution. Regarding the older horses, a possible explanation could be an increasing immunity that is not based on the antibodies detectable in the saliva-based ELISA. If immunity increases with age of the host, it might be able to suppress the infection burden to a lower level than that observed in more susceptible horses (about 20 worms/horse).

An increasing strongyle FEC was also associated with significantly higher odds for antibody detection in the saliva-based ELISA, with an increased odd of 1.01 for a positive test result for every increase by 100 of the epg. Similar results were reported by Jürgenschellert et al. [1] for the plasma-based ELISA. One explanation for this could be access to pasture, as this would be necessary for both infections. Other possible causes could be poor pasture hygiene or other management factors that increase contamination of the pasture with faeces.

Regular or partial paddock hygiene measures were also shown to be associated with significantly lower odds for positive tapeworm results in the bivariable model. Removing horse manure should reduce the risk of infection on the pasture as the eggs are removed along with the faeces, thus preventing them from being taken up by the intermediate hosts.

A partial or regular change of the pasture resulted in higher odds in the bivariable model in comparison to the constant use of the same pasture. This was also found in the study of Jürgenschellert et al. [1]. Removal of faeces was performed less frequently when paddocks were changed compared to yards where paddocks were never changed, which might explain this unexpected result. When horses return to previously contaminated paddocks, regrown grass may coincide with sufficient time for oribatid mites to harbour infective cysticercoids, facilitating completion of the tapeworm life cycle and increasing transmission risk.

Horses treated with praziquantel prior to sample collection had approximately 50% lower odds of testing positive in the saliva-based ELISA, although the difference was not statistically significant. These findings likely reflect current field practices, where most yards and horse owners treat tapeworm infections without first assessing for the presence of infection, typically administering strategic treatments to all horses once per year. This approach warrants reconsideration, particularly given the recently suggested reduction of treatment effectiveness of praziquantel and pyrantel against equine tapeworms from the US [35]. With no alternative tapeworm treatments available, diagnostic-led approaches to control should be implemented to reduce the risk of colic associated with pathogenic infections [18, 19] while limiting unnecessary treatments that drive anthelmintic resistance. Importantly, testing should be combined with effective management measures that reduce environmental contamination and transmission, ensuring fewer horses require treatment based on test results. Regular monitoring, when integrated with enhanced paddock hygiene practices, facilitates evidence-based interventions, preserves the efficacy of anthelmintics and will safeguard equine health over the long term.

## Conclusions

This study showed that antibody testing detects equine infections with Anoplocephalidae more effectively than the Mini-FLOTAC method, making it a valuable tool for screening and guiding anthelmintic treatment decisions. The most significant risk factor identified for a positive tapeworm saliva result was the origin: horses from Brandenburg and Saxony had significantly higher odds of testing positive than those from Schleswig-Holstein. Increasing FEC for strongyles in faecal analysis also had higher odds of tapeworm positivity, underscoring the impact of pasture access and management on infections with both of these types of parasites. Age was also found to have an effect: foals (< 1 year) and adults (> 5 years) had lower odds of tapeworm saliva test positivity compared to horses aged 1–5 years, highlighting the increased risk of disease-causing tapeworm burdens in the 1–5 year age group. Overall, the study demonstrated the combined influence of management, immunity and environmental exposure on tapeworm infection risk and supported the combined use of antibody testing and coproscopic examination to inform evidence-based control of this parasite.

## Supplementary Information

Additional File 1.

Additional File 2.

Additional File 3.

Additional File 4.

## Data Availability

All data supporting the findings of this study are available within the paper and its Supplementary Information.
