# Supplementary material for: High proportion of horses in Germany positive for antibodies against Anoplocephalidae
Source: Parasit Vectors. 2026 Oct 28;19:463. doi: 10.1186/s13071-026-07731-4 (PMC13718100; doi:10.1186/s13071-026-07731-4)
Supplement: Supplementary file 1 [file 13071_2026_7731_MOESM1_ESM.docx]

**Additional file 1: Text S1. Questionnaire**

**Questionnaire for research study**

**„Prevalence of gastro-intestinal nematodes in German stables “**

I. Keeping conditions
   1. Yard type:

☐Riding stable (less than 2 foals, more than 15 horses)

☐Small stable (less than 2 foals, less than 15 horses)

☐Breeding stable (more than 2 foals)

2. Total number of horses:
3. Age of horses (number per group):
   1) Foals (under 1 year):
   2) Young horses (1-5 Years):
   3) Adults (over 5 years):
4. Forms of husbandry:

☐Box

☐Loose housing (playpen)

☐Paddock boxes

☐Open stable

☐other:

II. Pasture management
    1. Do the horses have access to the pasture?	☐yes	☐no
    2. If yes, how long are the horses out in the pasture each day?

☐up to 4 hours	☐ up to 8 hours	☐over 8hours

3. How many hectares of pasture does the yard have:
4. Are pastures regularly changed?

☐yes	☐no

If yes, at what intervals?

5. Do you change the pastures after deworming?

☐yes	☐no

III. Hygiene management of stable and pasture
     1. How often do you clean the stables?
     2. Do you disinfect the stables?

☐yes	☐no

If yes how often and which disinfectant is used?

3. Is cleaning or desinfection carried out after deworming?

☐yes	☐no

4. Is feces collect from the pastures?
5. ☐yes	☐no

If yes, how often?

6. Is feces collected from the pastures?

☐yes	☐no

If yes, how often?

7. Are the pastures fertilised?

☐yes	☐no

If so, are they fertilized with manure?	☐yes	☐no

8. Do you use hay racks?

☐yes	☐no

IV. Deworming management
    1. Do you deworm regular?

☐yes	☐no (continue under 7.)

2. If yes, how often per year?

☐once	☐ twice	☐thrice	☐four times	☐other………………

3. Do you deworm all horses at the same time?	☐yes	☐no
4. Date of the last deworming?
5. Which drug was used?

Foals:

Young horses:

Adults:

Breeding mares:

6. Do you change the drug class regularly?	☐yes	☐no

If yes, how often?

7. Are new horses dewormed?	☐yes	☐no

Are fecal samples taken to monitor the success of the treatment?

8. Is deworming carried out selective after a fecal examination?

☐yes	☐no

If yes, at what level of infestation do you treat (fecal egg count)??

foals (under 1 year):…………………………..

young horses (1-5 years):……………………

Adults (over 5 years):…………………………..

9. Do you monitor the success of the anthelmintic treatment with fecal examinations?

☐yes	☐no

10. Please describe your anthelmintic management:

V. Stock health
   1. Have you recently observed the following symptoms in your yard? Emaciation, diarrhea, coughing, recurrent colic, poor performance?

☐yes	☐no

If yes, which symptoms?

2. Have you ever had problems with parasitic diseases in your yard?

☐yes	☐no

If yes, with which pathogen and clinical picture?

Comments:

Thank you for your participation!
