# Supplementary material for: High proportion of horses in Germany positive for antibodies against Anoplocephalidae
Source: Parasit Vectors. 2026 Oct 28;19:463. doi: 10.1186/s13071-026-07731-4 (PMC13718100; doi:10.1186/s13071-026-07731-4)
Supplement: Supplementary file 2 [file 13071_2026_7731_MOESM2_ESM.docx]

**Additional file 2: Tab. S1.** Proportions of positive test results according to saliva- and plasma-based ELISA on yard-level

| Yard number | Total number of horses in yard | Number of saliva samples | Proportion of positive horses - saliva ELISA (95% CI^a^) | Number of plasma samples | Proportion of positive horses - plasma ELISA (95% CI) |
| --- | --- | --- | --- | --- | --- |
| 1 | 10 | 9 | 33.3 (30.0 - 40.0) | 8 | 25.0 (20 - 0-40.0) |
| 2 | 76 | 10 | 20.0 (2.6 - 54.0) | 10 | 0 (0 - 30.3) |
| 3 | 32 | 11 | 54.6 (28.1 - 81.3) | 11 | 18.2 (6.3 - 46.9) |
| 4 | 80 | 10 | 10.0 (1.3 - 43.8) | 10 | 0 (0 - 30.0) |
| 5 | 40 | 10 | 60.0 (27.5 - 87.5) | 10 | 20.0 (5.0 - 52.5) |
| 6 | 34 | 10 | 50.0 (20.6 - 79.4) | 10 | 40.0 (14.7 - 70.6) |
| 7 | 56 | 10 | 80.0 (46.4 - 96.4) | 10 | 40.0 (12.5 - 71.4) |
| 8 | 9 | 9 | 100 | 9 | 88.9 |
| 9 | 60 | 10 | 50.0 (20.0 - 80.0) | 10 | 30.0 (6.7 - 63.3) |
| 10 | 20 | 10 | 60.0 (30.0 - 80.0) | 10 | 20.0 (10.0 - 50.0) |
| 11 | 10 | 10 | 20.0 | 10 | 0 |
| 12 | 55 | 8 | 50.0 (16.4 - 83.6) | 8 | 25.0 (3.6 - 63.6) |
| 13 | 66 | 10 | 20.0 (3.0 - 54.6) | 10 | 10.0 (1.5 - 42.4) |
| 14 | 60 | 10 | 60.0 (28.3 - 86.7) | 10 | 0 (0 - 30.0) |
| 15 | 42 | 12 | 8.3 (2.4 - 35.7) | 12 | 0 (0 - 23.8) |
| 16 | 75 | 10 | 10.0 (1.4 - 42.7) | 10 | 10.0 (1.3 - 42.7) |
| 17 | 26 | 10 | 70.0 (38.5 - 88.5) | 10 | 20.0 (7.7 - 50.0) |
| 18 | 40 | 10 | 30.0 (7.5 - 62.5) | 10 | 10.0 (2.5 - 42.5) |
| 19 | 22 | 12 | 75.0 (50.0 - 86.4) | 12 | 41.7 (22.7 - 68.2) |
| 20 | 36 | 10 | 10.0 (2.8 - 41.7) | 10 | 20.0 (5.6 - 52.8) |
| 21 | 43 | 10 | 0 (0 - 27.9) | 10 | 0 (0 - 27.9) |
| 22 | 18 | 10 | 20.0 (11.1 - 50.0) | 10 | 0 (0 - 22.2) |
| 23 | 50 | 10 | 0 (0 - 28.0) | 10 | 0 (0 - 28.0) |
| 24 | 28 | 10 | 30.0 (10.7 - 60.7) | 10 | 10.0 (3.6 - 39.3) |
| 25 | 65 | 10 | 60.0 (27.7 - 87.7) | 10 | 10.0 (1.5 - 43.1) |
| 26 | 120 | 6 | 16.7 (0.8 - 63.3) | 8 | 0 (0 - 36.7) |
| 27 | 60 | 10 | 10.0 (1.7 - 43.3) | 10 | 10.0 (1.7 - 43.3) |
| 28 | 50 | 9 | 44.4 (14.0 - 78.0) | 10 | 20.0 (4.0 - 54.0) |
| 29 | 39 | 10 | 0 (0 - 28.2) | 10 | 0 (0 - 28.2) |
| 30 | 80 | 9 | 0 (0 - 32.5) | 11 | 0 (0 - 27.5) |
| 31 | 68 | 12 | 50.0 (22.1 - 77.9) | 12 | 33.3 (10.3 - 63.2) |
| 32 | 37 | 10 | 0 (0 - 27.0) | 10 | 10.0 (2.7 - 40.5) |
| 33 | 38 | 9 | 11.1 (2.6 - 44.7) | 10 | 10.0 (2.6 - 42.1) |
| 34 | 25 | 11 | 72.7 (44.0 - 93.4) | 12 | 50.0 (24.0 - 73.0) |
| 35 | 23 | 11 | 9.1 (4.4 - 34.8) | 11 | 0 (0 - 21.7) |
| 36 | 31 | 9 | 11.1 (3.2 - 45.2) | 10 | 10.0 (3.2 - 41.9) |
| 37 | 24 | 10 | 10.0 (4.2 - 37.5) | 10 | 10.0 (4.2 - 37.5) |
| 38 | 37 | 10 | 30.0 (8.1 - 62.2) | 10 | 0 (0 - 27.0) |
| 39 | 60 | 10 | 80.0 (46.7 - 96.7) | 10 | 60.0 (28.3 - 86.7) |
| 40 | 60 | 12 | 0 (0 - 25.0) | 12 | 0 (0 - 25.0) |
| 41 | 18 | 7 | 0 (0 - 33.3) | 9 | 0 (0 - 27.8) |
| 42 | 104 | 10 | 0 (0 - 29.8) | 10 | 0 (0 - 29.8) |
| 43 | 55 | 8 | 12.5 (1.8 - 50.9) | 10 | 0 (0 - 29.1) |
| 44 | 28 | 10 | 0 (0 - 28.6) | 10 | 0 (0 - 28.6) |
| 45 | 40 | 10 | 0 (0 - 27.5) | 10 | 0 (0 - 27.5) |
| 46 | 60 | 9 | 33.3 (8.3 - 68.3) | 9 | 22.2 (3.3 - 58.3) |
| 47 | 10 | 10 | 10.0 | 10 | 0 |
| 48 | 19 | 9 | 0 (0 - 26.3) | 1 | n.a. |
| 49 | 10 | 10 | 10.0 | 10 | 0 |
| 50 | 22 | 10 | 0 (0 - 27.3) | 10 | 0 (0 - 27.3) |
| 51 | 35 | 9 | 11.1 (2.9 - 45.7) | 10 | 0 (0 - 28.6) |
| 52 | n.a. | 9 | 0 (n.a.) | 9 | n.a. |
| 53 | 24 | 10 | 0 (0 - 25.0) | 10 | 0 (0 - 25.0) |
| 54 | 60 | 10 | 10.0 (1.7 - 43.3) | 10 | 0 (0 - 30.0) |
| 55 | 200 | 11 | 0 (0 - 28.0) | 11 | 0 (0 - 28.0) |
| 56 | 40 | 10 | 0 (0 - 27.5) | 10 | 0 (0 - 27.5) |
| 57 | 24 | 9 | 22.2 (8.3 - 54.2) | 10 | 20.0 (8.3 - 50.0) |
| 58 | 18 | 9 | 11.1 (5.6 - 38.9) | 10 | 10.0 (5.6 - 38.9) |
| 59 | 23 | 10 | 0 (0 - 26.1) | 10 | 0 (0 - 26.1) |
| 60 | 20 | 10 | 0 (0 - 25.0) | 10 | 0 (0 - 25.0) |
| 61 | 36 | 10 | 0 (0 - 27.8) | 10 | 0 (0 - 27.8) |
| 62 | 18 | 10 | 0 (0 - 22.2) | 10 | 0 (0 - 22.2) |
| 63 | 100 | 8 | 0 (0 - 36.0) | 8 | 0 (0 - 36.0) |
| 64 | 43 | 10 | 10.0 (2.3 - 41.9) | 10 | 0 (0 - 27.9) |
| 65 | 29 | 10 | 0 (0 - 27.6) | 10 | 0 (0 - 27.6) |
| 66 | 53 | 8 | 0 (0 - 35.9) | 10 | 0 (0 - 28.3) |
| 67 | 65 | 10 | 0 (0 - 29.2) | 10 | 0 (0 - 29.2) |
| 68 | 42 | 10 | 10.0 (2.4 - 42.9) | 10 | 0 (0 - 28.6) |
| 69 | 93 | 11 | 18.2 (2.2 - 50.5) | 11 | 18.2 (2.2 - 50.5) |
| 70 | 40 | 12 | 16.7 (5.0 - 45.0) | 12 | 8.3 (2.5 - 35.0) |
| 71 | 180 | 10 | 20.0 (2.2 - 55.0) | 10 | 10.0 (0.6 - 43.9) |
| 72 | 31 | 10 | 0 (0 - 29.0) | 10 | 0 (0 - 29.0) |
| 73 | 22 | 10 | 0 (0 - 27.3) | 10 | 0 (0 - 27.3) |
| 74 | 10 | 10 | 0 | 10 | 0 |
| 75 | 45 | 9 | 11.1 (2.2 - 46.7) | 9 | 11.1 (2.2 - 46.7) |
| 76 | 14 | 10 | 10.0 (7.1 - 35.7) | 10 | 10.0 (7.1 - 35.7) |
| 77 | 25 | 8 | 0 (0 - 32.0) | 10 | 10.0 (4.0 - 40.0) |
| 78 | 43 | 10 | 0 (0 - 27.9) | 10 | 0 (0 - 27.9) |
| 79 | 22 | 10 | 70.0 (40.9 - 86.4) | 9 | 44.4 (18.2 - 72.7) |
| 80 | 37 | 9 | 20.0 (5.4 - 51.4) | 10 | 10.0 (2.7 - 40.5) |
| 81 | 30 | 10 | 30.0 (10.0 - 60.0) | 10 | 20.0 (6.7 - 53.3) |
| 82 | 10 | 10 | 70.0 | 10 | 60.0 |
| 83 | 85 | 9 | 44.4 (14.1 - 77.7) | 10 | 20.0 (2.4 - 54.1) |
| 84 | 42 | 10 | 0 (0 - 28.6) | 10 | 0 (0 - 28.6) |
| 85 | 36 | 8 | 0 (0 - 33.3) | 10 | 0 (0 - 27.8) |
| 86 | 38 | 10 | 0 (0 - 29.0) | 12 | 0 (0 - 23.7) |
| 87 | 100 | 9 | 0 (0 - 33.0) | 10 | 0 (0 - 30.0) |
| 88 | 43 | 10 | 30.0 (7.0 - 62.8) | 10 | 10.0 (2.3 - 41.9) |
| 89 | 40 | 11 | 9.1 (2.5 - 37.5) | 11 | 18.2 (5.0 - 47.5) |
| 90 | 76 | 10 | 50.0 (19.7 - 80.3) | 10 | 40.0 (13.2 - 72.4) |
| 91 | 35 | 10 | 0 (0 - 28.6) | 11 | 9.1 (2.9 - 37.1) |
| 92 | 17 | 10 | 0 (0 - 23.5) | 10 | 0 (0 - 23.5) |
| 93 | 29 | 7 | 0 (0 - 37.9) | 10 | 0 (0 - 27.6) |
| 94 | 18 | 9 | 0 (0 - 27.8) | 9 | 0 (0 - 27.8) |
| 95 | 10 | 8 | 0 (0 - 20.0) | 10 | 0 |
| 96 | 32 | 10 | 20.0 (6.3 - 53.1) | 10 | 40.0 (15.6 - 71.9) |

n.a. = not applicable

CI^a^ = confidence interval
