# Supplementary material for: High proportion of horses in Germany positive for antibodies against Anoplocephalidae
Source: Parasit Vectors. 2026 Oct 28;19:463. doi: 10.1186/s13071-026-07731-4 (PMC13718100; doi:10.1186/s13071-026-07731-4)
Supplement: Supplementary file 3 [file 13071_2026_7731_MOESM4_ESM.docx]

**Additional file 4: Text S2.** R script for data analysis

#Read data

setwd("Home/Dokuments")

library(readxl)

Data_table_publication <- read_excel("Data_table_publication.xlsx")

View(Data_table_publication)

#Load packages

library(geepack)

library(broom)

library(data.table)

#meadow_cleaning_new

#Remove rows with wissing values

Data_table_publication_table <- copy(Data_table_publication)

setDT(Data_table_publication_table)

typeof(Data_table_publication_table)

Data_table_publication_withoutNAs <- na.omit(Data_table_publication_table, cols=c("meadow_cleaning_new", "Anopl_Saliva"))

#Estimate generalized estimating equations

GEE_binomial_saliva_Ano_meadow_cleaning_new <- geeglm(Anopl_Saliva ~ as.factor(meadow_cleaning_new), data = Data_table_publication_withoutNAs, id = as.factor(Yard_number), family = binomial, corstr = "independence")

#Write estimates to tibble

GEE_binomial_saliva_Ano_meadow_cleaning_new_estimates <-

tidy(GEE_binomial_saliva_Ano_meadow_cleaning_new, conf.int = TRUE)

#Write odds ratios to tibble

GEE_binomial_saliva_Ano_meadow_cleaning_new_odds <-

tidy(GEE_binomial_saliva_Ano_meadow_cleaning_new, conf.int = TRUE, exponentiate = TRUE)

#Write data to file

write.csv2(GEE_binomial_saliva_Ano_meadow_cleaning_new_estimates, file = "GEE_binomial_saliva_Ano_meadow_cleaning_new_estimates.csv")

write.csv2(GEE_binomial_saliva_Ano_meadow_cleaning_new_odds, file = "GEE_binomial_saliva_Ano_meadow_cleaning_new_odds.csv")

#age group

#Estimate generalized estimating equations

GEE_binomial_saliva_Ano_age_group <- geeglm(Anopl_Saliva ~ relevel(as.factor(age_group), ref ="2"), data = Data_table_publication_withoutNAs, id = as.factor(Yard_number), family = binomial, corstr = "independence")

#Write estimates to tibble

GEE_binomial_saliva_Ano_age_group_estimates <-

tidy(GEE_binomial_saliva_Ano_age_group, conf.int = TRUE)

#Write odds ratios to tibble

GEE_binomial_saliva_Ano_age_group_odds <-

tidy(GEE_binomial_saliva_Ano_age_group, conf.int = TRUE, exponentiate = TRUE)

#Write data to file

write.csv2(GEE_binomial_saliva_Ano_age_group_estimates, file = "GEE_binomial_saliva_Ano_age_group_estimates.csv")

write.csv2(GEE_binomial_saliva_Ano_age_group_odds, file = "GEE_binomial_saliva_Ano_age_group_odds.csv")

#change_of_meadow_ordered

#Remove rows with wissing values

Data_table_publication_withoutNAs <- na.omit(Data_table_publication_table, cols=c("change_of_meadow_ordered", "Anopl_Saliva"))

#Estimate generalized estimating equations

GEE_binomial_saliva_Ano_change_of_meadow_ordered <- geeglm(Anopl_Saliva ~ relevel(as.factor(change_of_meadow_ordered), ref ="0"), data = Data_table_publication_withoutNAs, id = as.factor(Yard_number), family = binomial, corstr = "independence")

#Write estimates to tibble

GEE_binomial_saliva_Ano_change_of_meadow_ordered_estimates <-

tidy(GEE_binomial_saliva_Ano_change_of_meadow_ordered, conf.int = TRUE)

#Write odds ratios to tibble

GEE_binomial_saliva_Ano_change_of_meadow_ordered_odds <-

tidy(GEE_binomial_saliva_Ano_change_of_meadow_ordered, conf.int = TRUE, exponentiate = TRUE)

#Write data to file

write.csv2(GEE_binomial_saliva_Ano_change_of_meadow_ordered_estimates, file = "GEE_binomial_saliva_Ano_change_of_meadow_ordered_estimates.csv")

write.csv2(GEE_binomial_saliva_Ano_change_of_meadow_ordered_odds, file = "GEE_binomial_saliva_Ano_change_of_meadow_ordered_odds.csv")

#drug_against_tapeworms_PYR

#Remove rows with wissing values

Data_table_publication_withoutNAs <- na.omit(Data_table_publication, cols=c("drug_against_tapeworm_PYR", "Anopl_Saliva"))

#Estimate generalized estimating equations

GEE_binomial_saliva_Ano_drug_against_tapeworm_PYR <- geeglm(Anopl_Saliva ~ relevel(as.factor(drug_against_tapeworm_PYR), ref ="None"), data = Data_table_publication_withoutNAs, id = as.factor(Yard_number), family = binomial, corstr = "independence")

#Write estimates to tibble

GEE_binomial_saliva_Ano_drug_against_tapeworm_PYR_estimates <-

tidy(GEE_binomial_saliva_Ano_drug_against_tapeworm_PYR, conf.int = TRUE)

#Write odds ratios to tibble

GEE_binomial_saliva_Ano_drug_against_tapeworm_PYR_odds <-

tidy(GEE_binomial_saliva_Ano_drug_against_tapeworm_PYR, conf.int = TRUE, exponentiate = TRUE)

#Write data to file

write.csv2(GEE_binomial_saliva_Ano_drug_against_tapeworm_PYR_estimates, file = "GEE_binomial_saliva_Ano_drug_against_tapeworm_PYR_estimates.csv")

write.csv2(GEE_binomial_saliva_Ano_drug_against_tapeworm_PYR_odds, file = "GEE_binomial_saliva_Ano_drug_against_tapeworm_PYR_odds.csv")

#federal_states Schleswig_Holstein as reference

#Remove rows with wissing values

Data_table_publication_withoutNAs <- na.omit(Data_table_publication_table, cols=c("federal_state", "Anopl_Saliva"))

#Estimate generalized estimating equations

GEE_binomial_saliva_Ano_federal_state <- geeglm(Anopl_Saliva ~ relevel(as.factor(federal_state), ref ="Schleswig_Holstein"), data = Data_table_publication_withoutNAs, id = as.factor(Yard_number), family = binomial, corstr = "independence")

#Write estimates to tibble

GEE_binomial_saliva_Ano_federal_state_estimates <-

tidy(GEE_binomial_saliva_Ano_federal_state, conf.int = TRUE)

#Write odds ratios to tibble

GEE_binomial_saliva_Ano_federal_state_odds <-

tidy(GEE_binomial_saliva_Ano_federal_state, conf.int = TRUE, exponentiate = TRUE)

#Write data to file

write.csv2(GEE_binomial_saliva_Ano_federal_state_estimates, file = "GEE_binomial_saliva_Ano_federal_state_estimates.csv")

write.csv2(GEE_binomial_saliva_Ano_federal_state_odds, file = "GEE_binomial_saliva_Ano_federal_state_odds.csv")

#fertilization_with_feces

#Remove rows with wissing values

Data_table_publication_withoutNAs <- na.omit(Data_table_publication_table, cols=c("fertalization_with_feces", "Anopl_Saliva"))

#Estimate generalized estimating equations

GEE_binomial_saliva_Ano_fertalization_with_feces <- geeglm(Anopl_Saliva ~ relevel(as.factor(fertalization_with_feces), ref ="0"), data = Data_table_publication_withoutNAs, id = as.factor(Yard_number), family = binomial, corstr = "independence")

#Write estimates to tibble

GEE_binomial_saliva_Ano_fertalization_with_feces_estimates <-

tidy(GEE_binomial_saliva_Ano_fertalization_with_feces, conf.int = TRUE)

#Write odds ratios to tibble

GEE_binomial_saliva_Ano_fertalization_with_feces_odds <-

tidy(GEE_binomial_saliva_Ano_fertalization_with_feces, conf.int = TRUE, exponentiate = TRUE)

#Write data to file

write.csv2(GEE_binomial_saliva_Ano_fertalization_with_feces_estimates, file = "GEE_binomial_saliva_Ano_fertalization_with_feces_estimates.csv")

write.csv2(GEE_binomial_saliva_Ano_fertalization_with_feces_odds, file = "GEE_binomial_saliva_Ano_fertalization_with_feces_odds.csv")

#Meadow_time

#Remove rows with wissing values

Data_table_publication_withoutNAs <- na.omit(Data_table_publication_table, cols=c("Meadow_time", "Anopl_Saliva"))

#Estimate generalized estimating equations

GEE_binomial_saliva_Ano_Meadow_time <- geeglm(Anopl_Saliva ~ relevel(as.factor(Meadow_time), ref ="3"), data = Data_table_publication_withoutNAs, id = as.factor(Yard_number), family = binomial, corstr = "independence")

#Write estimates to tibble

GEE_binomial_saliva_Ano_Meadow_time_estimates <-

tidy(GEE_binomial_saliva_Ano_Meadow_time, conf.int = TRUE)

#Write odds ratios to tibble

GEE_binomial_saliva_Ano_Meadow_time_odds <-

tidy(GEE_binomial_saliva_Ano_Meadow_time, conf.int = TRUE, exponentiate = TRUE)

#Write data to file

write.csv2(GEE_binomial_saliva_Ano_Meadow_time_estimates, file = "GEE_binomial_saliva_Ano_Meadow_time_estimates.csv")

write.csv2(GEE_binomial_saliva_Ano_Meadow_time_odds, file = "GEE_binomial_saliva_Ano_Meadow_time_odds.csv")

#times_of_deworming

#Remove rows with wissing values

Data_table_publication_withoutNAs <- na.omit(Data_table_publication_table, cols=c("times_of_deworming", "Anopl_Saliva"))

#Estimate generalized estimating equations

GEE_binomial_saliva_Ano_times_of_deworming <- geeglm(Anopl_Saliva ~ relevel(as.factor(times_of_deworming), ref ="1"), data = Data_table_publication_withoutNAs, id = as.factor(Yard_number), family = binomial, corstr = "independence")

#Write estimates to tibble

GEE_binomial_saliva_Ano_times_of_deworming_estimates <-

tidy(GEE_binomial_saliva_Ano_times_of_deworming, conf.int = TRUE)

#Write odds ratios to tibble

GEE_binomial_saliva_Ano_times_of_deworming_odds <-

tidy(GEE_binomial_saliva_Ano_times_of_deworming, conf.int = TRUE, exponentiate = TRUE)

#Write data to file

write.csv2(GEE_binomial_saliva_Ano_times_of_deworming_estimates, file = "GEE_binomial_saliva_Ano_times_of_deworming_estimates.csv")

write.csv2(GEE_binomial_saliva_Ano_times_of_deworming_odds, file = "GEE_binomial_saliva_Ano_times_of_deworming_odds.csv")

#metric variables

#size (Horses per yard)

#Remove rows with wissing values

Data_table_publication_withoutNAs <- na.omit(Data_table_publication_table, cols=c("size", "Anopl_Saliva"))

#Estimate generalized estimating equations

GEE_binomial_saliva_Ano_size <- geeglm(Anopl_Saliva ~ size, data = Data_table_publication_withoutNAs, id = as.factor(Yard_number), family = binomial, corstr = "independence")

#Write estimates to tibble

GEE_binomial_saliva_Ano_size_estimates <-

tidy(GEE_binomial_saliva_Ano_size, conf.int = TRUE)

#Write odds ratios to tibble

GEE_binomial_saliva_Ano_size_odds <-

tidy(GEE_binomial_saliva_Ano_size, conf.int = TRUE, exponentiate = TRUE)

#Write data to file

write.csv2(GEE_binomial_saliva_Ano_size_estimates, file = "GEE_binomial_saliva_Ano_size_estimates.csv")

write.csv2(GEE_binomial_saliva_Ano_size_odds, file = "GEE_binomial_saliva_Ano_size_odds.csv")

#size_meadow_ha

#Remove rows with wissing values

Data_table_publication_withoutNAs <- na.omit(Data_table_publication_table, cols=c("size_meadow_ha", "Anopl_Saliva"))

#Estimate generalized estimating equations

GEE_binomial_saliva_Ano_size_meadow_ha <- geeglm(Anopl_Saliva ~ size_meadow_ha, data = Data_table_publication_withoutNAs, id = as.factor(Yard_number), family = binomial, corstr = "independence")

#Write estimates to tibble

GEE_binomial_saliva_Ano_size_meadow_ha_estimates <-

tidy(GEE_binomial_saliva_Ano_size_meadow_ha, conf.int = TRUE)

#Write odds ratios to tibble

GEE_binomial_saliva_Ano_size_meadow_ha_odds <-

tidy(GEE_binomial_saliva_Ano_size_meadow_ha, conf.int = TRUE, exponentiate = TRUE)

#Write data to file

write.csv2(GEE_binomial_saliva_Ano_size_meadow_ha_estimates, file = "GEE_binomial_saliva_Ano_size_meadow_ha_estimates.csv")

write.csv2(GEE_binomial_saliva_Ano_size_meadow_ha_odds, file = "GEE_binomial_saliva_Ano_size_meadow_ha_odds.csv")

#Strongyle EPG/100

#Remove rows with wissing values

Data_table_publication_withoutNAs <- na.omit(Data_table_publication_table, cols=c("Epg_gastro_intestinal_strongyles", "Anopl_Saliva"))

#Estimate generalized estimating equations

GEE_binomial_saliva_Ano_Epg_gastro_intestinal_strongyles <- geeglm(Anopl_Saliva ~ I(Epg_gastro_intestinal_strongyles/100), data = Data_table_publication_withoutNAs, id = as.factor(Yard_number), family = binomial, corstr = "independence")

#Write estimates to tibble

GEE_binomial_saliva_Ano_Epg_gastro_intestinal_strongyles_estimates <-

tidy(GEE_binomial_saliva_Ano_Epg_gastro_intestinal_strongyles, conf.int = TRUE)

#Write odds ratios to tibble

GEE_binomial_saliva_Ano_Epg_gastro_intestinal_strongyles_odds <-

tidy(GEE_binomial_saliva_Ano_Epg_gastro_intestinal_strongyles, conf.int = TRUE, exponentiate = TRUE)

#Write data to file

write.csv2(GEE_binomial_saliva_Ano_Epg_gastro_intestinal_strongyles_estimates, file = "GEE_binomial_saliva_Ano_Epg_gastro_intestinal_strongyles_estimates.csv")

write.csv2(GEE_binomial_saliva_Ano_Epg_gastro_intestinal_strongyles_odds, file = "GEE_binomial_saliva_Ano_Epg_gastro_intestinal_strongyles_odds.csv")

#Remove rows with wissing values

Data_table_publication_withoutNAs <- na.omit(Data_table_publication_table, cols=c("Epg_gastro_intestinal_strongyles", "times_of_deworming", "Meadow_time", "federal_state", "drug_against_tapeworm", "meadow_cleaning_new", "age_group", "Anopl_Saliva"))

#Estimate generalized estimating equations

GEE_binomial_saliva_Ano_Multi1 <- geeglm(Anopl_Saliva ~ Epg_gastro_intestinal_strongyles + relevel(as.factor(times_of_deworming), ref ="1") + relevel(as.factor(Meadow_time), ref ="3") + relevel(as.factor(federal_state), ref = "Schleswig_Holstein") + relevel(as.factor(drug_against_tapeworm), ref = "None") + relevel(as.factor(meadow_cleaning_new), ref ="0") + relevel(as.factor(age_group), ref ="2"), data = Data_table_publication_withoutNAs, id = as.factor(Yard_number), family = binomial, corstr = "independence")

#Write estimates to tibble

GEE_binomial_saliva_Ano_Multi1_estimates <-

tidy(GEE_binomial_saliva_Ano_Multi1, conf.int = TRUE)

#Write odds ratios to tibble

GEE_binomial_saliva_Ano_Multi1_odds <-

tidy(GEE_binomial_saliva_Ano_Multi1, conf.int = TRUE, exponentiate = TRUE)

#Write data to file

write.csv2(GEE_binomial_saliva_Ano_Multi1_estimates, file = "GEE_binomial_saliva_Ano_Multi1_estimates.csv")

write.csv2(GEE_binomial_saliva_Ano_Multi1_odds, file = "GEE_binomial_saliva_Ano_Multi1_odds.csv")

#Remove rows with wissing values

Data_table_publication_withoutNAs <- na.omit(Data_table_publication_table, cols=c("Epg_gastro_intestinal_strongyles", "Meadow_time", "federal_state", "drug_against_tapeworm", "meadow_cleaning_new", "age_group", "Anopl_Saliva"))

#Estimate generalized estimating equations

GEE_binomial_saliva_Ano_Multi2 <- geeglm(Anopl_Saliva ~ Epg_gastro_intestinal_strongyles + relevel(as.factor(Meadow_time), ref ="3") + relevel(as.factor(federal_state), ref = "Schleswig_Holstein") + relevel(as.factor(drug_against_tapeworm), ref = "None") + relevel(as.factor(meadow_cleaning_new), ref ="0") + relevel(as.factor(age_group), ref ="2"), data = Data_table_publication_withoutNAs, id = as.factor(Yard_number), family = binomial, corstr = "independence")

#Write estimates to tibble

GEE_binomial_saliva_Ano_Multi2_estimates <-

tidy(GEE_binomial_saliva_Ano_Multi2, conf.int = TRUE)

#Write odds ratios to tibble

GEE_binomial_saliva_Ano_Multi2_odds <-

tidy(GEE_binomial_saliva_Ano_Multi2, conf.int = TRUE, exponentiate = TRUE)

#Write data to file

write.csv2(GEE_binomial_saliva_Ano_Multi2_estimates, file = "GEE_binomial_saliva_Ano_Multi2_estimates.csv")

write.csv2(GEE_binomial_saliva_Ano_Multi2_odds, file = "GEE_binomial_saliva_Ano_Multi2_odds.csv")

#Remove rows with wissing values

Data_table_publication_withoutNAs <- na.omit(Data_table_publication_table, cols=c("Epg_gastro_intestinal_strongyles", "federal_state", "drug_against_tapeworm", "meadow_cleaning_new", "age_group", "Anopl_Saliva"))

#Estimate generalized estimating equations

GEE_binomial_saliva_Ano_Multi3 <- geeglm(Anopl_Saliva ~ Epg_gastro_intestinal_strongyles + relevel(as.factor(federal_state), ref = "Schleswig_Holstein") + relevel(as.factor(drug_against_tapeworm), ref = "None") + relevel(as.factor(meadow_cleaning_new), ref ="0") + relevel(as.factor(age_group), ref ="2"), data = Data_table_publication_withoutNAs, id = as.factor(Yard_number), family = binomial, corstr = "independence")

#Write estimates to tibble

GEE_binomial_saliva_Ano_Multi3_estimates <-

tidy(GEE_binomial_saliva_Ano_Multi3, conf.int = TRUE)

#Write odds ratios to tibble

GEE_binomial_saliva_Ano_Multi3_odds <-

tidy(GEE_binomial_saliva_Ano_Multi3, conf.int = TRUE, exponentiate = TRUE)

#Write data to file

write.csv2(GEE_binomial_saliva_Ano_Multi3_estimates, file = "GEE_binomial_saliva_Ano_Multi3_estimates.csv")

write.csv2(GEE_binomial_saliva_Ano_Multi3_odds, file = "GEE_binomial_saliva_Ano_Multi3_odds.csv")

#Remove rows with wissing values

Data_table_publication_withoutNAs <- na.omit(Data_table_publication_table, cols=c("Epg_gastro_intestinal_strongyles", "federal_state", "drug_against_tapeworm", "age_group", "Anopl_Saliva"))

#Estimate generalized estimating equations

GEE_binomial_saliva_Ano_Multi4 <- geeglm(Anopl_Saliva ~ Epg_gastro_intestinal_strongyles + relevel(as.factor(federal_state), ref = "Schleswig_Holstein") + relevel(as.factor(drug_against_tapeworm), ref = "None") + relevel(as.factor(age_group), ref ="2"), data = Data_table_publication_withoutNAs, id = as.factor(Yard_number), family = binomial, corstr = "independence")

#Write estimates to tibble

GEE_binomial_saliva_Ano_Multi4_estimates <-

tidy(GEE_binomial_saliva_Ano_Multi4, conf.int = TRUE)

#Write odds ratios to tibble

GEE_binomial_saliva_Ano_Multi4_odds <-

tidy(GEE_binomial_saliva_Ano_Multi4, conf.int = TRUE, exponentiate = TRUE)

#Write data to file

write.csv2(GEE_binomial_saliva_Ano_Multi4_estimates, file = "GEE_binomial_saliva_Ano_Multi4_estimates.csv")

write.csv2(GEE_binomial_saliva_Ano_Multi4_odds, file = "GEE_binomial_saliva_Ano_Multi4_odds.csv")

#Remove rows with wissing values

Data_table_publication_withoutNAs <- na.omit(Data_table_publication_table, cols=c("Epg_gastro_intestinal_strongyles", "federal_state", "age_group", "Anopl_Saliva"))

#Estimate generalized estimating equations

GEE_binomial_saliva_Ano_Multi5 <- geeglm(Anopl_Saliva ~ Epg_gastro_intestinal_strongyles + relevel(as.factor(federal_state), ref = "Schleswig_Holstein") + + relevel(as.factor(age_group), ref ="2"), data = Data_table_publication_withoutNAs, id = as.factor(Yard_number), family = binomial, corstr = "independence")

#Write estimates to tibble

GEE_binomial_saliva_Ano_Multi5_estimates <-

tidy(GEE_binomial_saliva_Ano_Multi5, conf.int = TRUE)

#Write odds ratios to tibble

GEE_binomial_saliva_Ano_Multi5_odds <-

tidy(GEE_binomial_saliva_Ano_Multi5, conf.int = TRUE, exponentiate = TRUE)

#Write data to file

write.csv2(GEE_binomial_saliva_Ano_Multi5_estimates, file = "GEE_binomial_saliva_Ano_Multi5_estimates.csv")

write.csv2(GEE_binomial_saliva_Ano_Multi5_odds, file = "GEE_binomial_saliva_Ano_Multi5_odds.csv")

#Remove rows with wissing values

Data_table_publication_withoutNAs <- na.omit(Data_table_publication_table, cols=c("Epg_gastro_intestinal_strongyles", "federal_state", "age_group", "Anopl_Saliva"))

#Estimate generalized estimating equations

GEE_binomial_saliva_Ano_Multi5_100 <- geeglm(Anopl_Saliva ~ I(Epg_gastro_intestinal_strongyles/100) + relevel(as.factor(federal_state), ref = "Schleswig_Holstein") + + relevel(as.factor(age_group), ref ="2"), data = Data_table_publication_withoutNAs, id = as.factor(Yard_number), family = binomial, corstr = "independence")

#Write estimates to tibble

GEE_binomial_saliva_Ano_Multi5_100_estimates <-

tidy(GEE_binomial_saliva_Ano_Multi5_100, conf.int = TRUE)

#Write odds ratios to tibble

GEE_binomial_saliva_Ano_Multi5_100_odds <-

tidy(GEE_binomial_saliva_Ano_Multi5_100, conf.int = TRUE, exponentiate = TRUE)

#Write data to file

write.csv2(GEE_binomial_saliva_Ano_Multi5_100_estimates, file = "GEE_binomial_saliva_Ano_Multi5_100_estimates.csv")

write.csv2(GEE_binomial_saliva_Ano_Multi5_100_odds, file = "GEE_binomial_saliva_Ano_Multi5_100_odds.csv")
